# Stability and flexibility in cognitive control: Interindividual dynamics and task context processing

**DOI:** 10.1371/journal.pone.0219397

**Published:** 2019-07-10

**Authors:** Deborah J. Serrien, Louise O’Regan

**Affiliations:** School of Psychology, University of Nottingham, Nottingham, United Kingdom; RWTH Aachen, GERMANY

## Abstract

Adaptive behaviour requires cognitive control for shielding current goals from distractors (stability) but at the same time for switching between alternative goals (flexibility). In this behavioural study, we examine the stability-flexibility balance in left- and right-handers during two types of decision-making, instructed (sensory cued) and voluntary (own choice), by means of distractor inhibition and hand/task switching. The data revealed that both groups showed opposite tendencies for instructed decision-making. Moreover, right-handers resisted distracting information more efficiently whereas left-handers showed superior switching abilities. When participants were involved in voluntary decision-making, no effects of handedness were noted, which suggests that free-choice processing alters the balance between stability and flexibility. These data illustrate that handedness is an index of individual variation during instructed decision-making, biasing the proficiency of cognitive control towards stability and flexibility of information processing. These biases can however be overruled by top-down strategies that dominate during voluntary decision-making. Overall, the research underlines the antagonistic functions of stability and flexibility in decision-making, and offers an approach for examining cognitive control and the role of internal and external factors in balancing the stability-flexibility trade-off.

## Introduction

In daily life, we often have to perform a task while ignoring distractions, or, switch between tasks in response to changing demands. In these cases, cognitive control supports our decision-making through two antagonistic modes; stability and flexibility, which respectively enable us to shield existing goals and to update them when needed [1–2]. Accordingly, there are complementary benefits and costs associated with both cognitive control modes. That is, while strong goal shielding facilitates behavioural persistence with top-down bias towards the current goal, it incurs a cost in terms of perseveration and reduced adaptation to variable requirements. Conversely, while weak goal shielding assists switching between alternatives and greater autonomy, it augments distractibility due to increased sensitivity to bottom-up influences [1]. From a computational perspective, stable states can be conceptualised as basins in a potential landscape. In such models, stable states correspond to working memory representations (goals) with the basin depth characterising the stability of the attractor such that deeper basins reflect more stable states that are less likely destabilised by distractions or perturbations, and that require more effort to switch behaviour [3]. The general principles of this framework closely link with the dynamic systems approach to motor control that underscores that behaviour reflects a balance between stability, instability, and flexibility [4] as evidenced by research examining motor control and coordination tasks [5–7].

Whereas cognitive stability can be studied through delayed response tasks in combination with the presentation of distractors, cognitive flexibility can be assessed in experimental paradigms that require switching between tasks, or, that involve shifting attention to different characteristics within a single task [8–9]. Assignments that involve distractor inhibition and task switching are usually associated with inferior performances, including slowed response times and increased error rates, as compared to control conditions. Thus, these particular situations are more time-consuming and/or more difficult, reflected by performance costs for inhibition and switching that capture the underlying cognitive control processes. In general, these types of settings are evaluated through forced-choice trials during which the participants receive instructions about the tasks to perform. However, an exception to these is voluntary task switching during which participants can choose which task to do on each trial, or, on an intermittent basis, enabling the computation of the voluntary switch rate that exemplifies cognitive flexibility during free-choice trials [10–12].

Research investigating the regulation of cognitive stability and flexibility has demonstrated a common frontoparietal network that supports adaptive information coding [13–14], albeit with lateral prefrontal cortices that are specialised for stability as opposed to parietal and frontolateral regions for flexibility [9,15]. Furthermore, the mechanisms that implement stability and flexibility also regulate their balance [16]. In particular, metacontrol refers to the ability to allocate cognitive resources to the actual demands and affects two parameters: the degree of competition between the alternatives and the degree to which this competition is biased by the current goal [2]. Noteworthy is that the trade-off between stability and flexibility is sensitive to individual factors such as personality traits [2,16]. However, individual differences do not necessarily emerge from maxima of cognitive control but rather from the efficient integration of information and change of strategy when needed.

There are various approaches for understanding individual differences [17]. In this work, we study the relevance of an essential functional characteristic; handedness, which reflects asymmetry for manual control and is guided by a dominant hand for skilled unimanual and bimanual activities [18]. Handedness is a multifactorial trait that is determined by multiple genetic and environmental factors, and that is characterised by functional as well as structural neural asymmetries [19]. It is acknowledged that left- and right-handers have distinct involvement of motor circuits within as well as between hemispheres with the former group showing augmented information communication between both sides [20–25]. However, less is known about how handedness influences cognitive control. Although, handedness is often linked with cognition, findings on this association are inconsistent and there is no consensus whether being left- or right-handed has an advantage for cognitive regulation [26–27]. Establishing cognitive efficiency as a function of handedness is important as it closely relates to managing everyday situations. This evaluation is not only relevant for decisions that are triggered externally by the environment (e.g., by sensory cue) but also those that are generated internally by the individual (i.e., own choice) as both types of tasks rely on different processing demands of top-down and bottom-up influences [28–29].

The study of individual differences is key for understanding distinct traits in cognitive domains. Accordingly, the aim of the present research is to investigate the balance between stability (to shield goals from distraction) and flexibility (update goals when needed) of information processing in left- and right-handers. The work will offer new insights into individual differences in cognitive control and the relationship with handedness. It is argued that handedness influences hemispheric engagement, leading to variation of cognitive performance with left-handers showing increased flexibility for dealing with the demands.

## Materials and methods

### Participants

A total of 42 participants were involved in this study. No psychiatric or neurological conditions as assessed by questionnaire were reported, and all participants had normal or corrected-to-normal vision. The study was approved by the ethics committee of the School of Psychology, University of Nottingham (Ethics Approval Number 604R). All participants provided written informed consent before starting data acquisition.

### Handedness questionnaire

The participants answered a questionnaire with 15 items about their preference for unimanual and bimanual activities (i.e., write letter, use scissors when cutting paper, use comb, hold racquet, use toothbrush, throw ball to hit a target, hold knife to cut, hold needle when seewing, use eraser on paper, deal cards, use spoon, peel apple, draw picture, use broom for sweeping, open lid from can). The questionnaire made use of a Likert scale. Moreover, the score per handedness item was computed as 0 = always left hand, 1 = usually left hand, 2 = both hands equally, 3 = usually right hand, and 4 = always right hand. For every participant, the scores of the handedness items were summed, and divided by the maximum score of the questionnaire, and multiplied by 100. Accordingly, the handedness score varied between 0 = extreme left-handedness and 100 = extreme right-handedness, and resulted in 21 left-handers (*M*_AGE_ = 22.38±0.85; *M*_HAND_ = 20.71±3.21) and 21 right-handers (*M*_AGE_ = 20.42±0.64; *M*_HAND_ = 92.10±1.55). Besides the handedness score, the writing hand was a requirement for the characterisation of handedness [30]. The family history of left-handedness (with at least one parent being a left-hander) was also examined and revealed eight left-handers (39%) and three right-handers (14%). The procedure was similar as described previously [31].

### Cognitive tasks

The participants were seated in front of a computer screen with a viewing distance of 70 cm. Variants of the same experimental paradigm were conducted under instructed (cued, forced-choice) and voluntary (internal, free-choice) conditions. There were three main cognitive tasks: (1) instructed inhibition, (2) instructed switching, and (3) voluntary switching. Across all, participants performed numerical judgment tasks, e.g., categorising numbers as odd vs. even, or, as greater vs. less than 5. During the instructed inhibition and switching tasks, participants responded to external cues whereas they made their own decision on an intermittent basis during the voluntary task [11]. The distinction allowed us to compare decision-making that involves an unambiguous mapping of explicit stimuli to task responses with decision-making that includes an arbitrary decision from the participants (although an implicit cue is presented to indicate the own choice requirement). The participants received practice trials to familiarise with the various cognitive tasks and breaks were provided during the study. The experimental designs were established by means of the PsychoPy software [32].

#### Instructed decision-making: Distractor-present and distractor-absent tasks

**Fig 1:**
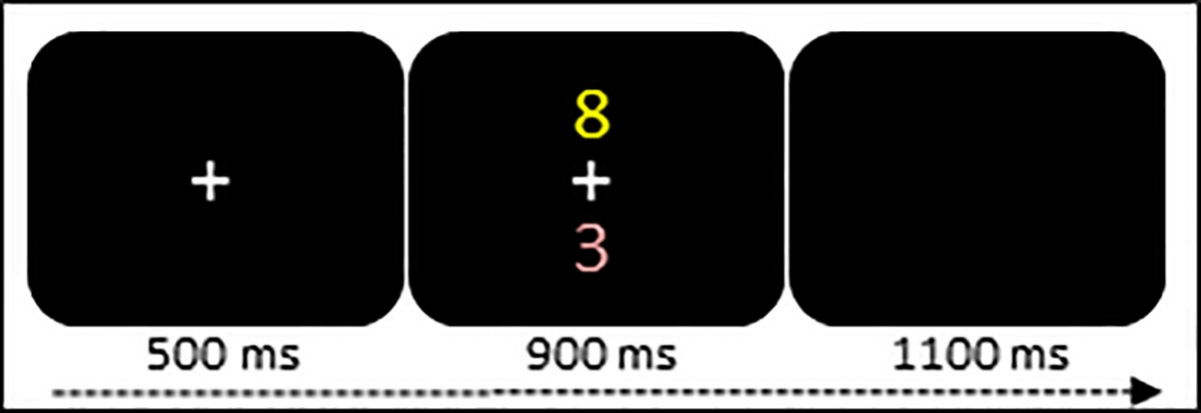
Schematic illustration of the instructed distractor inhibition task. After presentation of the fixation cross, a target number (yellow font) was presented on its own or in the presence of a distractor number (red font). Participants had to ignore the distractor number and respond to the target number.

The trial sequence is displayed in Fig 1. The trials had a fixed duration of 2500 ms, and consisted of the presentation of a fixation cross at the centre of the screen followed by a target number between 1 and 9 (excluding 5) that was presented in yellow font either above or below the fixation cross (distractor-absent trials). In some trials, a congruent distractor number presented in red font appeared simultaneously with the target number. The participants had to decide whether the target was odd or even, or, greater or less than 5, with both task rules being completed in separate trial blocks. The participants were instructed to respond as fast and as accurately as possible to the target by means of bimanual key presses and ignore the distractors that were irrelevant to the task. Both index fingers were used if the target was odd or greater than 5 whereas both middle fingers were used If the target was even or less than 5. There was a total of 200 trials, and the distractors were present alongside the targets in 25% of the trials.

#### Instructed decision-making: Repetition, task and hand switching tasks

**Fig 2:**
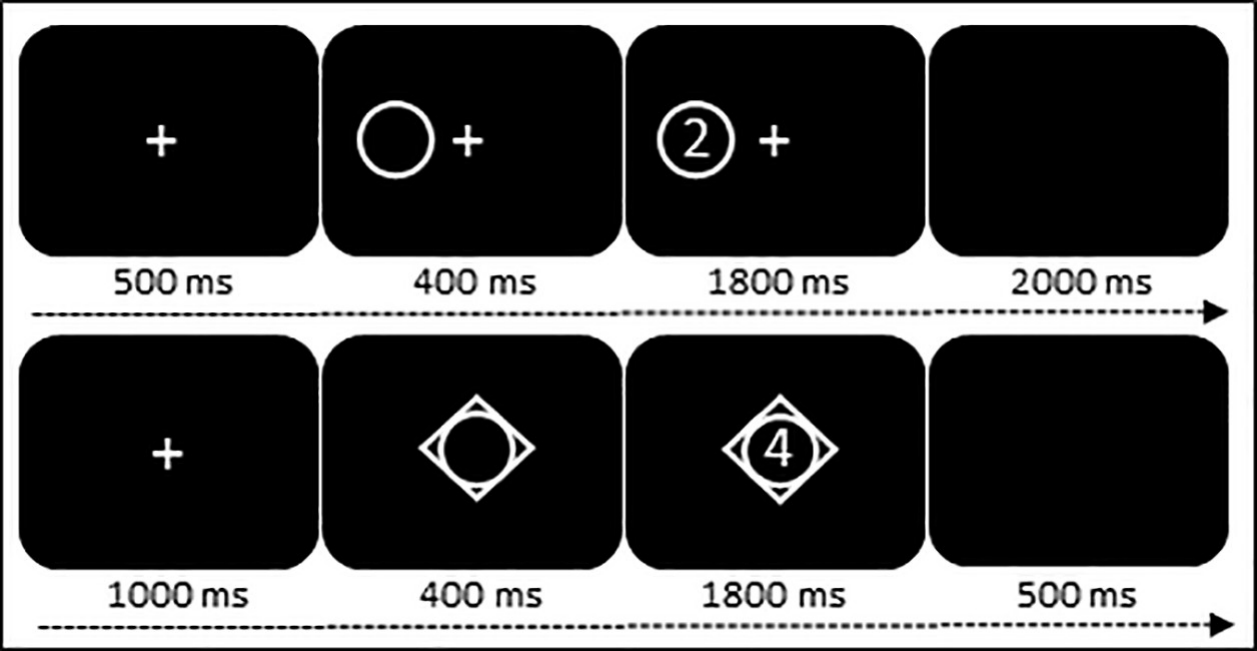
*Upper panel*: Schematic illustration of the instructed switching task. After presentation of the fixation cross, a cue was shown on the left or right side of the fixation cross to inform the participants with which hand to respond. The cue, which had the shape of a circle for the odd/even task, instructed the participants about the task to perform. Following, a target number was displayed within the borders of the cue. *Lower panel*: Schematic illustration of the voluntary switching task. After presentation of the fixation cross, a cue that had the overlaid shape of a diamond and circle was shown in case the participants had to make a voluntary decision about the task to perform.

The trial sequence is shown in Fig 2 (upper panel). Trials had a fixed duration of 4700 ms, and involved the presentation of a fixation cross at the centre of the screen followed by a cue that informed the participants which task to perform during the next trial. The cue had the shape of a circle for the odd/even task and a diamond shape for the greater/less than 5 task whereas its location, i.e., on the left or right side of the fixation cross, informed the participants whether to respond with the left or right hand. Subsequently, a target number was presented within the borders of the cue. The index finger of the left/right hand was used if the target was odd or greater than 5 whereas the middle finger of the left/right hand was used If the target was even or less than 5. There were three different types of trials: task repetition + hand repetition, task switch (with hand repetition), and hand switch (with task repetition). Therefore, the aim was to compare trials for which both task and hand were repetitions vs. trials for which one component switched while the other component remained unchanged. There was a total of 256 trials. On 50% of the trials, there were repetitions for both task and hand whereas the other 50% of the trials were switch trials. Of the switch trials, half of them were task switches (hand unchanged) whereas the other half were hand switches (task unchanged).

#### Voluntary decision-making: Repeat and switching tasks

The trial sequence is illustrated in Fig 2 (lower panel). Trials had a fixed duration of 3700 ms, and consisted of the presentation of a fixation cross at the centre of the screen followed by a cue that informed the participants which task to perform during the next trial. This cue had a circle shape for the odd/even task, a diamond shape for the greater/less than 5 task, and a diamond inside a circle shape for the voluntary task during which participants made a decision themselves about which task to do. The participants were told that they were free which task to perform during the free-choice trials, which could not repeat. The responses were mapped to different hands; the odd/even task with the left index/middle fingers, and greater/less than 5 task with the right index/middle fingers. There was a total of 144 trials with 33% of free-choice trials.

### Measurements

*First*, for instructed decision-making (distractor inhibition and switching tasks), the main behavioural measures of response time and response accuracy were used as indicators of cognitive processing that associate with forced-choice decisions. For the distraction inhibition task, the responses from the left and right key presses were averaged. The largest between-hand difference across participants was 30 ms. We further calculated the processing costs that related with the conditions of inhibition and switching. In particular, inhibition costs referred to the response times with distractor *minus* the response times without distractor whereas the switching costs represented the switching times *minus* the repetition times. *Second*, for voluntary decision-making, the main behavioural measures of response time and response rate (repeat, switch) were used as indicators of cognitive processing that link with free-choice decisions. The voluntary trials were labelled as a repeat/switch if the task on trial n was identical/different as the task on trial n– 1. For a number of voluntary trials (around 20%), participants pressed the response keys of both hands, or, provided no response so that no clear task choice could be identified. These trials were labelled as omissions and were not included in the analysis.

### Analysis

The statistical analyses involved mixed-design ANOVAs and t-tests. Post-hoc comparisons with Bonferroni corrections were made where necessary. Pearson’s correlations were conducted between the voluntary and instructed decision-making tasks to examine the overlap of computational processes. Mean±SE scores are reported. All scores were averaged over trials per performance condition. Only correct responses were analysed.

## Results

### Instructed decision-making: Distractor-present and distractor-absent tasks

The response times and response accuracy scores were analysed using mixed 2 × 2 ANOVAs (Handedness Group: left- vs. right-handers; Task Condition: no distractor vs. with distractor). The two distractor inhibition tasks (odd/even and greater/less than 5) were averaged for the ANOVA analyses as the processing costs of distractor-present vs. distractor-absent conditions did not reveal significant differences *p* > 0.05.

#### Response times

**Fig 3:**
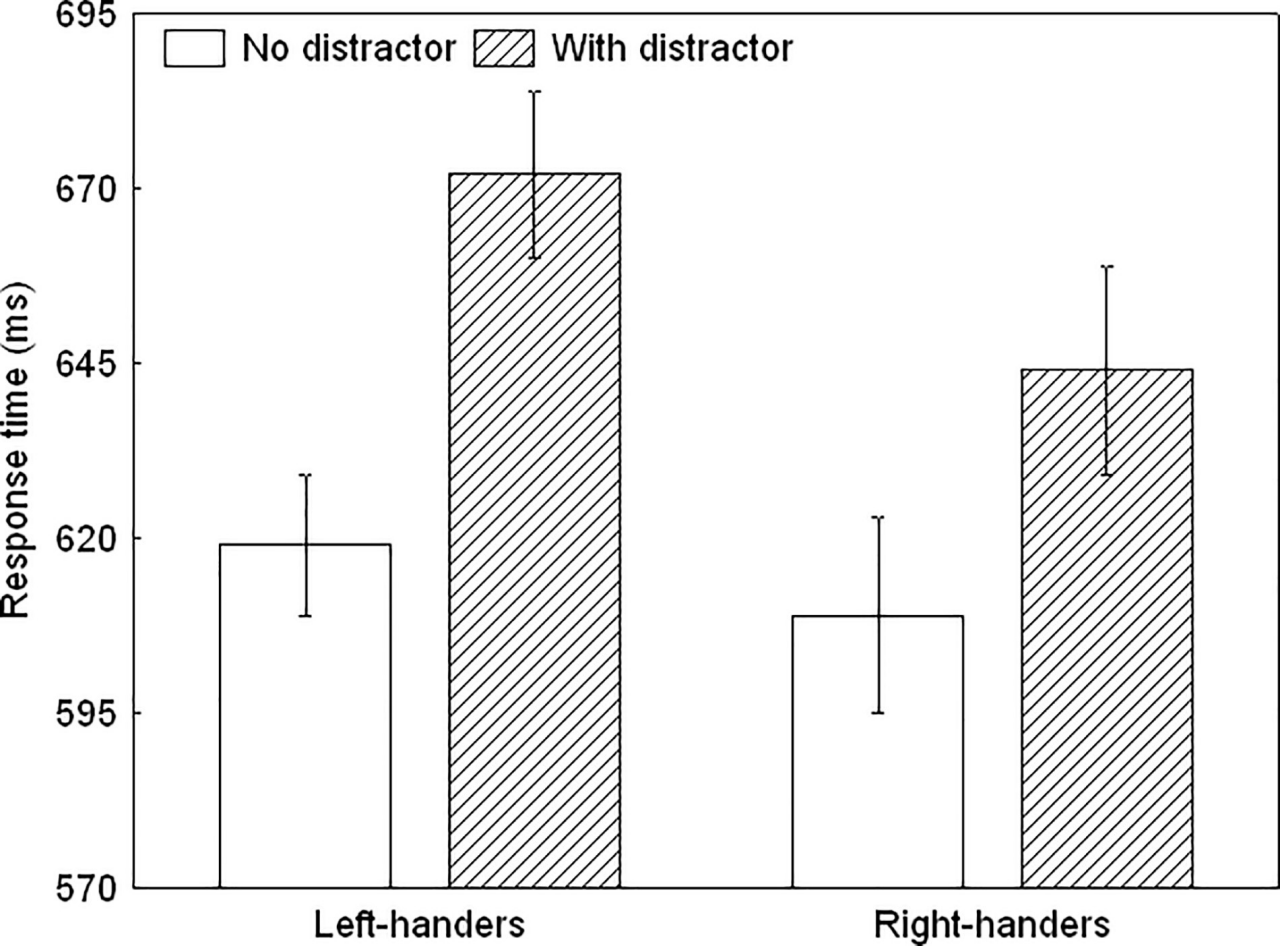
The response times for the instructed distractor inhibition task for the left- and right-handers. Left-handers were less successful than right-handers for responding to the target when a distractor was present.

The ANOVA revealed a significant main effect of Task Condition, *F*(1,40) = 103.41, *p* < 0.01, ηp^2^ = 0.72 as well as a significant Handedness Group x Task Condition interaction, *F*(1,40) = 4.71, *p* < 0.05, ηp^2^ = 0.11 (Fig 3). Left- and right-handers did not differ significantly for both task conditions (*p* > 0.05). However, the inhibition costs, which exemplifies the difference between the distractor-present and distractor-absent trials, were significantly larger for the left- than right-handers, *t*(40) = 2.16, *p* < 0.05.

#### Response accuracy

The ANOVA showed a significant main effect of Task Condition, *F*(1,40) = 57.05, *p* < 0.01, ηp^2^ = 0.59, and indicated that responding with distractor (84±1%) was less accurate than without distractor (89±1%).

### Instructed decision-making: Repetition, task and hand switching tasks

The response times and response accuracy scores were analysed using mixed 2 × 3 ANOVAs (Handedness Group: left- vs. right-handers; Task Condition: repetition vs. hand switch vs. task switch).

#### Response times

**Fig 4:**
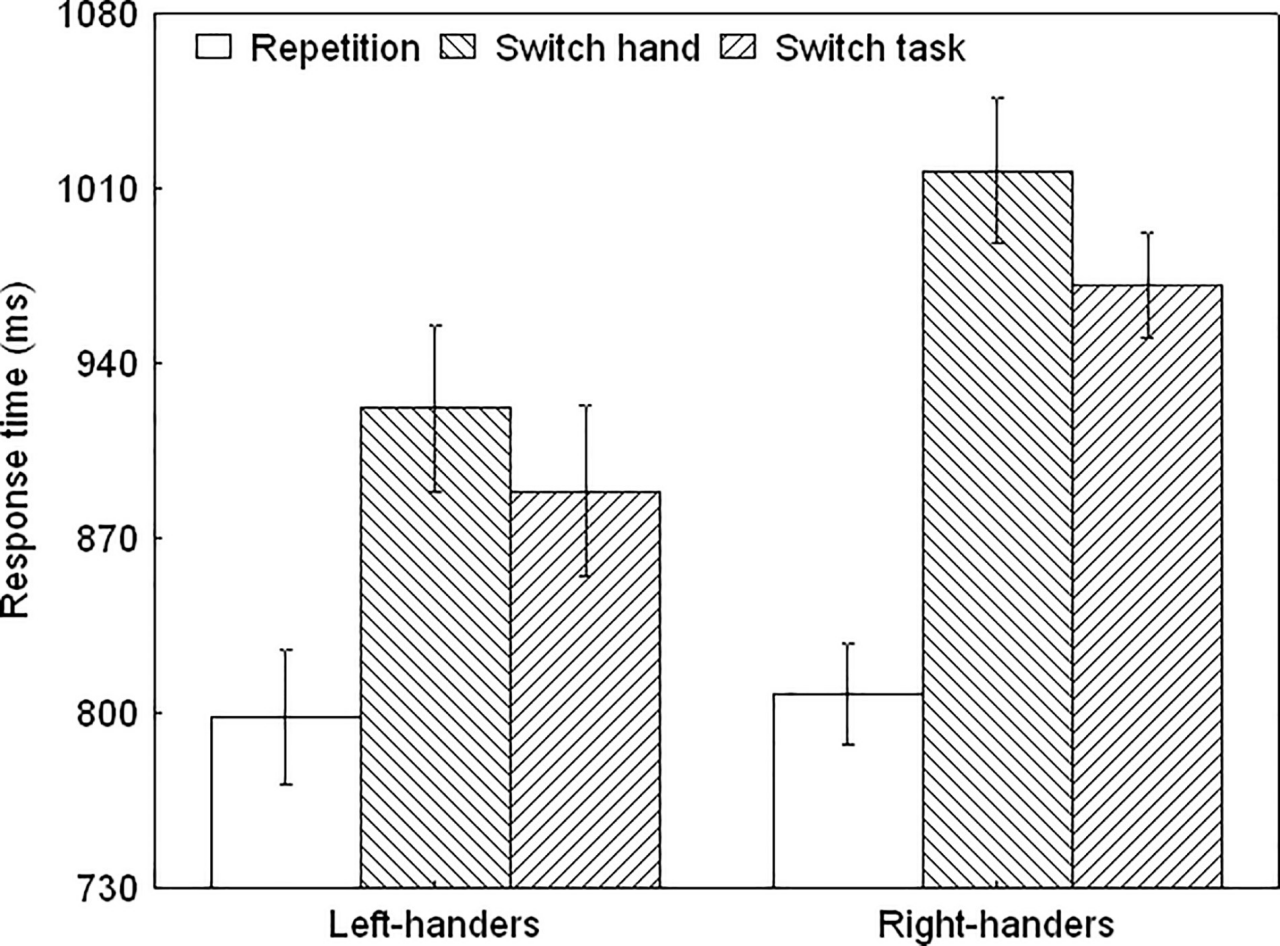
The response times for the instructed switching tasks for the left- and right-handers. Left-handers were more efficient than right-handers when switching to an alternative goal was required.

The ANOVA demonstrated a significant main effect of Task Condition, *F*(1,40) = 56.09, *p* < 0.01, ηp^2^ = 0.58. The Handedness Group x Task Condition interaction was also significant, *F*(2,80) = 3.85, *p* < 0.05, ηp^2^ = 0.12 (Fig 4). There were no differences between the groups in the repetition condition (*p* > 0.05) whereas left-handers were faster than right-handers in the hand and task switching conditions (*p* < 0.05, for both). Furthermore, the switching costs, which depicts the difference between the switch and the repeat trials, were significantly smaller for the left- than right-handers during hand switching, *t*(40) = 2.55, *p* < 0.05, and task switching *t*(40) = 2.54 *p* < 0.05.

#### Response accuracy

The ANOVA revealed a significant main effect of Task Condition, *F*(2,80) = 24.19, *p* < 0.01, ηp^2^ = 0.45. Post-hoc comparisons revealed that task conditions differed from one another. In particular, highest accuracy was achieved in the repetition condition followed by the task and hand switching conditions; 85±2%; 80±2% and 71±2%, *p* ≤ 0.05.

### Voluntary decision-making: Repeat and switching tasks

The response times were analysed using mixed 2 × 2 ANOVA (Handedness Group: left- vs. right-handers; Task Condition: repeat vs. switch).

#### Response times

The ANOVA demonstrated a significant main effect of Task Condition, *F*(1,40) = 9.26, *p* < 0.01, ηp^2^ = 0.17, and reflected faster response times for repeats (880±30 ms) than for switches (937±31 ms). No other effects were significant, *p* > 0.05.

#### Stability-flexibility association

**Fig 5:**
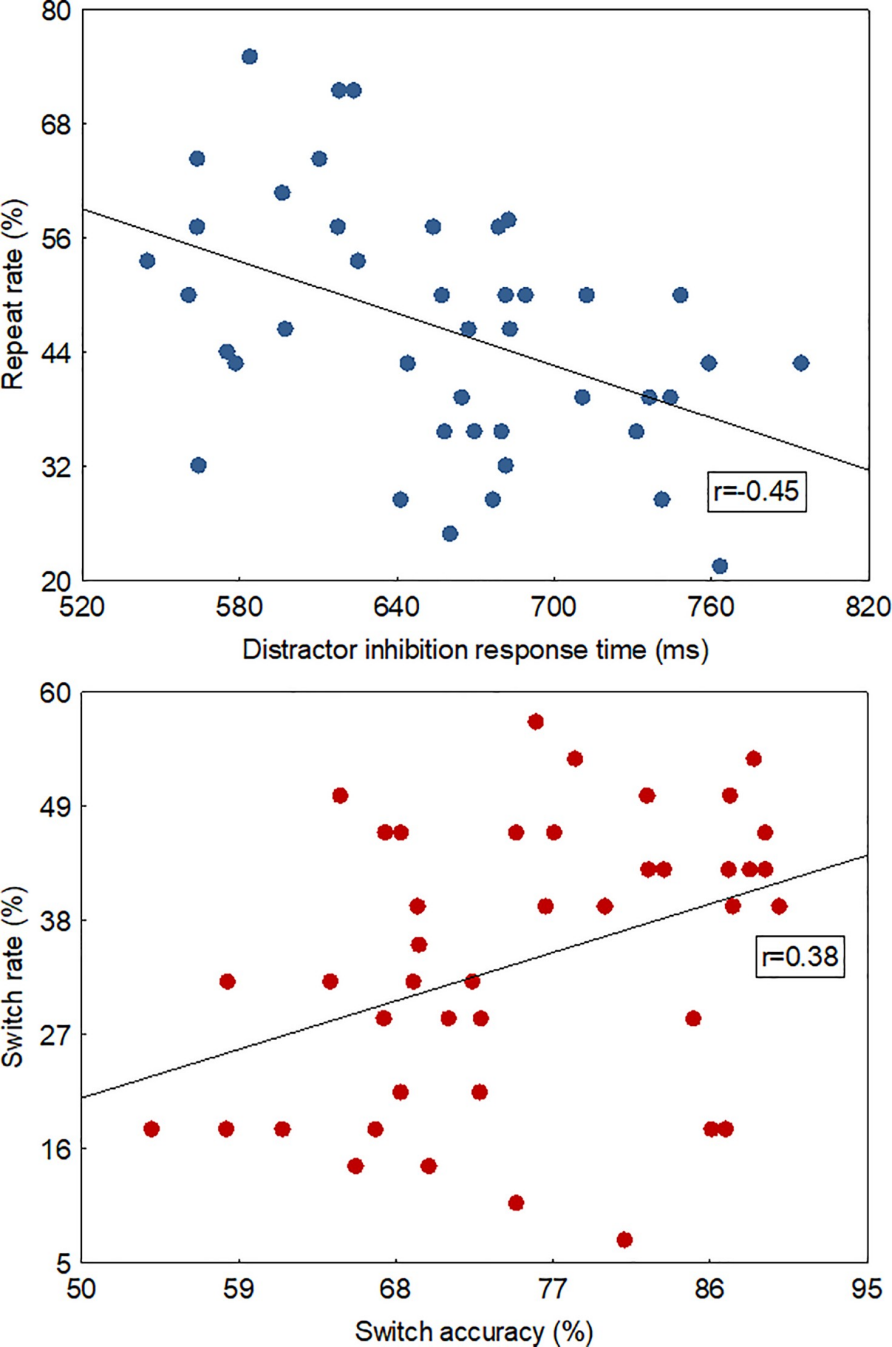
(top) A scatter plot between the voluntary repeat rate (46±2%) and instructed distractor inhibition response time across handedness groups. It shows that higher repeat rates in the voluntary task associated with faster cued inhibition response times.lower) A scatter plot between the voluntary switch rate (34.±2%) and instructed switch accuracy (combined for hand and task switches) across handedness groups. It depicts that higher switch rates in the voluntary task associated with higher cued switching accuracy.

Correlation analyses were conducted to establish the computational overlap between voluntary and instructed responses. A significant negative correlation between the repeat rate and cued distractor inhibition response time was observed (*r* = -0.45, *p* < 0.01, Fig 5A). No significant association between the repeat rate and cued distractor inhibition accuracy was noted, *p* > 0.05. A significant positive correlation between the switch rate and cued switching accuracy was revealed (*r* = 0.38, *p* < 0.05, Fig 5B). No significant association was revealed between the switch rate and cued switching response time, *p* > 0.05.

## Discussion

Adaptive goal-directed behaviour requires cognitive control and comprises stability (i.e., to maintain goals and shield them from distractors) and flexibility (i.e., to relax goals and update them). Furthermore, the ability to allocate cognitive resources in given situations relies on a mechanism that balances between extreme forms of stability and flexibility of information processing, also referred to as metacontrol [2]. In this study, we investigated the impact of individual differences on the proficiency of stability and flexibility demands. In particular, we explored how left- and right-handers differ in their ability to adjust cognitive control during distractor inhibition and switching conditions.

## Instructed decision-making and its effect on stability and flexibility

Cognitive control involves a dynamic trade-off between stability and flexibility [1–2]. Here, we adopted a framework that assessed both control modes within a comparable experimental context. To study stability, we used an inhibition task that measured goal maintenance in the presence of distracting information whereas flexibility was studied by means of a switching task that measured the implementation of a shift towards a new goal. Typically, these types of experimental paradigms use visual cues that instruct participants to continue, or, to change the current task set.

During instructed inhibition, participants were slower and less accurate in responding to a target during distractor-present vs. distractor-absent trials. This suggests that focus on the goal requirements was affected by the distracting stimulus, resulting in inhibition costs due to target-distractor competition and interference. Various factors make a stimulus distracting such as perceptual salience [33], and proximity to the target or shared characteristics [34–35]. It has been proposed that increased activation in frontoparietal regions captures the processing demands in the face of competing internal or external demands [36] with in particular right frontal circuitry dealing with distractor challenges and mediating interactions between top-down and bottom-up influences [37–38]. However, our data also showed that the impact of the distractors on behaviour was not similar across handedness group. That is, left-handers were less efficient than right-handers in responding to the target during distractor-present trials, suggesting that handedness affects the proficiency of selective information processing and the disregard of irrelevant input.

During instructed switching, participants performed slower and were less accurate during switch as compared to repetition trials, in line with the presence of switching costs [29, 39–41]. Switch trials are therefore more complex than repetition trials as they usually involve additional cognitive processes [40]. Various factors influence the degree of switching costs such as preparation or predictability [42–43] and pre-cueing or practice [39–40]. Our data extend previous research by showing differences between the switching conditions. Moreover, switching of hand only was less successful than switching of task only, which suggests increased processing costs for implementing a change of effector. It has been suggested that switching associates with strengthened frontoparietal activation for updating goals at different representational levels [44–46]. Yet, we also observed that the effects of switching were distinct for both handedness groups. In particular, left-handers were more proficient than right-handers when changing the goal demands, suggesting that handedness influences the effectiveness of shifting between alternatives.

### Voluntary decision-making and its processing costs

In everyday situations, decisions are often made on the basis of internal goals rather than external cues from the environment. In this particular case, people favour the current goal as a reference for evaluating the (dis)advantages of the existing option and the alternatives [47]. In order to capture this type of decision-making that relies on internal choice, experimental paradigms can be used in which participants are free to select which task to perform as opposed to instructed switching that requires participants to adhere to external prompts or cues from the environment [10, 48]. Moreover, voluntary paradigms capture insights into executive control processes that are not represented in instructed paradigms as the absence of an explicit cue imposes a decision about whether to repeat or switch [28]. Thus, both types of tasks can be operationalised on the basis of dominant endogenous vs. exogenous influences for decision-making. We observed that participants were faster during repeats than switches, which underlines increased time-consuming processes for switching than repetition when decisions are based on free-choice [49].

According to a computational model of flexibility-stability, individual differences in attractor stability (i.e., depth of attractor basin) can be measured by using the voluntary rates as behavioural indexes. We observed that individuals with higher voluntary repeat rates were faster during cued inhibition trials, which denotes stronger goal shielding and an ability to resist distractions. Conversely, individuals with higher voluntary switch rates were more accurate during cued switching trials, indicating an increased efficiency for task updating and an ability to promote the achievement of new goals. Combined, these individual differences reveal that voluntary rates characterise a dynamic interplay of the flexibility-stability balance and capture a unique way of assessing executive control. The distinct effect on the measurements of timing and accuracy hints at dissociable influences between interacting mechanisms of decision-making [50].

### Handedness and individual differences in cognitive control

The study of handedness allows us to detail the processing mechanisms in different population groups. In this work, participants were categorised as a left- or right-hander by means of a self-report questionnaire [18, 31, 51]. Previously, performance differences as a function of handedness have been linked with distinct communication pathways across hemispheres and/or access to the right hemisphere. Moreover, intensified interhemispheric interactions in left- as compared to right-handers exist between the motor cortices as well as between the prefrontal regions that mediate cognitive processes [25, 52–53].

Our data highlighted that left- and right-handers showed different performance tendencies during instructed decision-making, suggesting that handedness shapes cognitive regulation. That is, whereas right-handers demonstrated increased goal maintenance and reduced distractor interference (stability), left-handers had an advantage for the relaxation of task shielding and switching between goals (flexibility). That group behaviour was modified in opposite ways indicates that the processing mechanisms that support handedness influence the relationship between stability and flexibility. Moreover, these findings relate with variation in cognitive control and behaviour between individuals as well as between performance conditions [2]. Here, the hypothesis is made that right- as compared to left-handers were more efficient during cued distractor inhibition, possibly due to reduced interhemispheric interactions that decreased the impact of the right hemisphere, which has a prime role in distractor processing and the integration of top-down and bottom-up information [36, 54]. Conversely, the hemispheres must interact extensively during switch trials [9], which likely enabled left- as compared to right-handers to obtain improved efficiency for cued switching due to stronger interhemispheric communication pathways. Therefore, handedness guides the ability to recruit cognitive control during instructed task demands.

Handedness did not affect the performance of voluntary trials, proposing that the processing mechanisms are distinct when people are in charge over their decisions as compared to when instructed by cues. In this respect, task reconfiguration vs. memory retrieval has been particularly associated with voluntary and instructed decisions, respectively [55]. This suggests that active strategies during voluntary decision-making alter the stability-flexibility balance and the impact of individual differences. Therefore, the data demonstrate that voluntary choices and the time taken to implement those decisions reflect complex interactions between different processing mechanisms that accordingly influence behaviour. Taking into account that most behaviour is situated on a continuum that lies between voluntary and instructed decisions, it means that a combination of top-down and bottom-up influences guide individual performance as a function of handedness.

In conclusion, the present findings illustrate that handedness is an index of individual variation during instructed decision-making, biasing the proficiency of cognitive control towards stability and flexibility of information processing. These biases can however be overruled by top-down strategies that dominate during voluntary decisions. The data further underline the antagonistic functions of stability and flexibility in decision-making, and offer an approach for examining cognitive control and the role of internal and external factors in balancing the stability-flexibility trade-off.
